# Characteristics of Patients With Atherosclerotic Cardiovascular Disease in Belgium and Current Treatment Patterns for the Management of Elevated LDL‐C Levels

**DOI:** 10.1002/clc.24330

**Published:** 2024-08-29

**Authors:** Eléonore Maury, Samuel Brouyère, Mieke Jansen

**Affiliations:** ^1^ Medical Department Novartis Pharma Vilvoorde Belgium; ^2^ Cegedim Health Data Belgium Brussels Belgium

**Keywords:** atherosclerotic cardiovascular disease, general practitioners, high cardiovascular risk, lipid‐lowering therapy, real‐world evidence

## Abstract

**Background:**

Dyslipidemia remains the major cause of atherosclerotic cardiovascular disease (ASCVD). Lipid management in patients with increased cardiovascular (CV) risk needs improvement across Europe, and data gaps are noticeable at the country level.

**Hypothesis:**

We described the current treatment landscape in Belgium, hypothesizing that lipid management in patients with ASCVD remains inadequate and aiming to understand the reasons.

**Methods:**

Using data from an anonymized primary care database in Belgium derived from 494 750 individuals, we identified those with any CV risk factor between November 2019 and October 2022 and described the clinical features of patients with ASCVD. The main outcomes were the proportion of patients (i) receiving lipid‐lowering therapies (LLTs), (ii) per low‐density lipoprotein cholesterol (LDL‐C) threshold, stratified per LLT, (iii) reaching the 2021 ESC recommended LDL‐C goals, and (iv) LDL‐C reduction per type of LLT was also determined.

**Results:**

Among 40 888 patients with very high CV risk, 24 859 had established ASCVD. Most patients with ASCVD were either receiving monotherapy (59.6%) or had no documented LLT (25.1%). Further, 64.2% of those with no documented LLT exhibited LDL‐C levels ≥ 100 mg/dL. Among common treatment options, one of the greatest improvements in LDL‐C levels was achieved with combination therapy of statin and ezetimibe, reducing LDL‐C levels by 41.5% (*p* < 0.0001). Yet, in this group, 24.8% of patients had still LDL‐C levels ≥ 100 mg/dL and only 20.7% were at goal.

**Conclusion:**

Our study emphasizes the importance of developing strategies to help patients achieve their LDL‐C goals, with a focus on supporting the implementation of combination LLT in routine clinical practice.

## Practitioner Points

- Approximately 25% of patients with ASCVD had no documented LLT (although 64.2% of them exhibited LDL‐C levels ≥ 100 mg/dL), ~60% received monotherapy, and only 15.3% received combination LLT.
- The greatest improvements in LDL‐C levels were achieved with high‐intensity statin plus ezetimibe. Yet, ~25% of patients receiving statin plus ezetimibe had LDL‐C levels ≥ 100 mg/dL, and only 20.7% were at goal (2021 ESC guidelines).
- Altogether, the results emphasize the importance of developing strategies to help patients achieve their LDL‐C goals, with a focus on supporting the implementation of combination LLT in routine clinical practice.

## 1 Introduction

Cardiovascular disease (CVD) is the leading cause of death and disability worldwide [1]. Atherosclerotic cardiovascular disease (ASCVD) is the major component of this disease [2], arising from the buildup of lipid‐containing atheroma in arterial walls [3, 4]. Reducing morbidity and mortality risk associated with ASCVD involves lowering low‐density lipoprotein cholesterol (LDL‐C) levels [4, 5, 6, 7], which may be achieved with lifestyle interventions, with maximum tolerated dose of statin and ezetimibe if required, and with a proprotein convertase subtilisin kexin 9 (PCSK9) inhibitor for patients unable to reach their LDL‐C target levels or statin or ezetimibe intolerant [2].

The DA VINCI study provided data on lipid‐lowering therapy (LLT) treatment patterns in Europe between June 2017 and November 2018 and revealed a large gap between the LDL‐C goals advocated by the 2019 ESC/EAS guidelines [5] and the LDL‐C levels achieved in routine clinical practice in Belgium [8, 9]. Less than half of patients (41%) reached their risk‐based LDL‐C goal, emphasizing the imperative for revised clinical approaches in dyslipidemia management in Belgium [9]. The SANTORINI study recently reported the lack of improvements in Europe over the past years, although not yet capturing the impact of lipid‐lowering medications recently approved by the European Medicines Agency (EMA) (i.e., bempedoic acid and PCSK9 inhibitors) [10]. This finding might be explained by CV risk underestimation, as only approximately half of physicians reported using the 2019 ESC/AES guidelines for risk classification, more than 20% of patients had no documented LLT, and combination therapies were underutilized [10]. Recent studies further highlight the poor awareness of CV risk factors and ASCVD in Belgian patients hospitalized for CVD [11, 12].

To the best of our knowledge, up‐to‐date numbers on the prevalence of ASCVD in Belgium are lacking to monitor the situation, have a better understanding of the disease burden, and define the best measures needed to manage the disease and lower its prevalence and burden [12, 13]. In this context, our study aims at defining the characteristics of patients with ASCVD in Belgium. This real‐world evidence study explores the burden of ASCVD by examining the CV risk factors measured in general practitioners' (GP) practices and provides insights into current treatment patterns.

## 2 Materials and Methods

### 2.1 Study Design and Data Sources

This was an observational, retrospective, noninterventional study with secondary use of data, extracted from anonymized patient health records, obtained from the Belgian version of The Health Improvement Network (THIN®, Cegedim Health Data). The THIN database obtained approval from the Belgian Data Protection Authority and has collected data since 2005. It consists of anonymized electronic medical records compliant with the European general data protection regulations. The Belgian legislation did not require additional ethical approval. The requirement for obtaining informed consent was waived.

### 2.2 Patients

All patients undergoing a medical examination at a GP practice between November 2019 and October 2022 were assessed for previous or current cardiometabolic disease and treatment with LLT. Out of 494 750 patients undergoing a medical examination at a GP practice between November 2019 and October 2022, all 283 700 patients with at least 1 year of historical data were included (File S1).

### 2.3 Variables

CV risk categories were defined at the patient level, as defined by the 2021 ESC Guidelines on CVD prevention in clinical practice [2], for those with at least one risk factor documented in the medical file between November 2019 and October 2022 (File S1). Systematic Coronary Risk Estimation 2 (SCORE2) was determined per age group, as previously defined [2], using individual patient values for blood pressure, non‐HDL‐C, and smoking status (i.e., evidence for smoking was collected for smoking cessation therapy) during this period. Evidence of target organ damage (TOD; retinopathy, nephropathy, and neuropathy) was examined [2]. ASCVD was considered present if the capture of any relevant ICD10 diagnoses [2] was reported in the medical files between November 2019 and October 2022.

Maximal CV risk (ever reached) and the transitions from moderate to high and very high CV risk were also determined per individual patient, based on the most severe status at any time during the recorded medical history since 2005. Hence, the period covered a timeframe of at least 1 year, as per inclusion rules, to 17 years (i.e., 2005). When referring to medical history for medications, anamnesis, diagnoses, or events, this period was also used.

When referring to current or latest treatments, all LLT treatments prescribed within the 1.5 years preceding the most recent LLT prescription data available in the patient file are described. Current or latest LDL‐C values are the latest values recorded in the patient files. Maximal LDL‐C (ever reached) was the highest LDL‐C value reported at any time during the recorded medical history since 2005. Baseline LDL‐C values refer to LDL‐C levels before pharmacological treatment initiation. Baseline and LDL‐C after LLT initiation both require a 1‐year observation period before the first prescription, allowing to confirm the first date of LLT prescription, at any point in the patient's observed history. The analyses of LDL‐C levels in all Figures (except for Figure S3) were performed after the exclusion of the minority of patients receiving PCSK9 inhibitors (< 50 patients). In some analyses, the patients are divided into subgroups based on commonly used LDL‐C goals [2], with, in some instances, an additional threshold of 130 mg/dL [14, 15, 16].

### 2.4 Statistical Analysis

Results were either presented descriptively (e.g., prevalence or percentage of patients receiving a specific treatment) or as mean ± standard error of the mean (SEM) for group comparison. Normality of data distribution was calculated, and two‐tailed paired Student's *t*‐test or Wilcoxon matched‐pair signed‐rank test were applied, as appropriate. Differences were considered statistically significant at *p* < 0.05, with *p < 0.05, **p < 0.01, ***p < 0.001, and ****p < 0.0001, using Prism v9.5.1 Software (GraphPad, La Jolla, CA, USA).

## 3 Results

Out of the total of 283 700 patients studied, there were 27 956 patients (9.9%) categorized as having low to moderate CV risk (Figure S1). Additionally, 40 867 patients (14.4%) were identified as having high CV risk, and there were 40 888 patients (14.4%) having very high CV risk, including individuals with established ASCVD (Figure S1). Among patients with low to moderate CV risk, 23.4% were diagnosed with diabetes for less than 10 years, with no evidence of TOD and no additional ASCVD risk factors while 76.7% were in this risk category based on SCORE2. Among patients with high CV risk, 4.3% had an estimated glomerular filtration rate (eGFR) between 30 and 44 mL/min/1.73 m^2^, 20.5% were diagnosed with diabetes for 10 years or more and 1.9% had evidence of TOD despite diabetes diagnosed for less than 10 years, and 74.3% had high CV risk based on SCORE2. Individuals with very high CV risk included 33.3% of patients above the highest SCORE2 cut‐offs, 4.1% with chronic kidney disease with eGFR < 30 mL/min/1.73 m^2^, and 5.5% of them were diagnosed with diabetes and concomitant eGFR < 45 mL/min/1.73 m^2^. This very high‐risk group also included 60.8% of patients with established ASCVD.

Based on the most severe status of these patients reported in the medical history, 15.5% were at low to moderate CV risk, 19.0% at high CV risk, and 14.4% at very high CV risk, according to the guidelines [2] (Figure S2). A total of 5.2% of the overall cohort transitioned from low‐moderate to high risk, 4.6% from high to very high risk but only 0.4% transitioned directly from low‐moderate to very high risk (Figure S2).

We focused on the 24 859 patients with established ASCVD in this database. Projecting this number to the estimated number of patients at the national scale, we evaluated that approximately 967 015 patients are living with ASCVD in Belgium (File S1).

The clinical and laboratory characteristics of these patients with ASCVD are depicted in Table 1. The mean (± SEM) age of the population was 72.5 ± 0.1 years, and there was a slight male preponderance (55.9%). The patients had a mean (± SEM) BMI of 29.0 ± 0.1 kg/m^2^, that is, falling within the overweight range. Although the history of antihypertensive treatment was very high in this population (93.1%), more than half of patients (57.0%) presented an elevated blood pressure (systolic ≥ 13 cm Hg and/or diastolic ≥ 8.5 cm Hg). Two‐thirds of the patients (66.9%) had renal disease. Most patients were diagnosed with coronary heart disease (65.2%), 36.9% with cerebrovascular disease, and 18.5% with peripheral artery disease, and the diagnosis was made, on average, > 13 years ago. More than one‐fourth (28.0%) of patients were previously diagnosed with Type 2 diabetes but only 26.3% received antidiabetic medications. Heterozygous familial hypercholesterolemia was found in 13.2% of patients (however, the Dutch Lipid Clinic Network score could not be retrieved). The percentage of patients with ASCVD and coexisting heart failure was 7.0%.

**Table 1:** Characteristics of patients with ASCVD.

	All patients with ASCVD	*N*
Age (years), mean ± SEM	72.5 ± 0.1	22 417
Sex, female, *n* (%)	10 916 (44.1)	24 779
Current clinical characteristics		
BMI (kg/m^2^), mean ± SEM	29.0 ± 0.1	8610
Systolic blood pressure (mmHg), mean ± SEM	127.2 ± 0.2	22 567
Diastolic blood pressure (mmHg), mean ± SEM	74.7 ± 0.2	22 407
High blood pressure, *n* (%)	12 779 (57.0)	22 407
eGFR (mL/min), mean ± SEM	74.1 ± 0.1	22 417
Renal disease (eGFR ≤ 89 mL/min/1.73 m^2^), *n* (%)	16 638 (66.9)	24 859
eGFR 60–89 mL/min/1.73 m^2^	10 876 (65.4)	16 638
eGFR 30–59 mL/min/1.73 m^2^	4959 (29.8)	16 638
eGFR < 30 mL/min/1.73 m^2^	803 (4.8)	16 638
Smoker, *n* (%)	2206 (8.9)	24 859
SCORE2, mean ± SEM	13.1 ± 0.1	13 560
Documented cardiovascular disease		
History of coronary heart disease (≥ 1 diagnosis), *n* (%)	16 219 (65.2)	24 859
History of cerebrovascular disease (≥ 1 diagnosis), *n* (%)	9183 (36.9)	24 859
History of peripheral artery disease (≥ 1 diagnosis), *n* (%)	4591 (18.5)	24 859
Visits related to an ASCVD diagnosis per patient, mean ± SEM	1.6 ± 0.0	24 859
Time since first diagnosis (days), mean ± SEM	4964.6 ± 18.2	24 859
Other comorbidities, *n* (%)		
Heterozygous familial hypercholesterolemia	3280 (13.2)	24 859
History of Type 2 diabetes	6955 (28.0)	24 859
Treatment with antidiabetic medication	6540 (26.3)	24 859
Treatment with antihypertensive medication	20 252 (93.1)	24 233
Liver disease	403 (1.6)	24 859
Heart failure	1727 (7.0)	24 859
NYHA Class I	576 (2.3)	24 859
NYHA Class II	745 (3.0)	24 859
NYHA Class III	361 (1.5)	24 859
NYHA Class IV	45 (0.2)	24 859
Lipids, mean ± SEM		
Latest total cholesterol (mg/dL)	172.8 ± 0.3	20 049
Latest LDL‐cholesterol (mg/dL)	93.8 ± 0.3	19 859
Maximum LDL‐cholesterol (mg/dL)	125.5 ± 0.3	19 859
Lipid‐lowering therapy		
History of LLT intake, *n* (%)	18 617 (74.9)	24 859
LLT treatment duration (days), mean ± SEM	2782.0 ± 14.8	18 617
Number of molecules used (*N*), mean ± SEM	1.6 ± 0.0	18 617
Number of up titrations (*N*), mean ± SEM	0.4 ± 0.0	18 617
Number of down titrations (*N*) mean ± SEM	0.2 ± 0.0	18 617
History of LLT treatment interruption, *n* (%)	7049 (37.9)	18 617
Statins^a^		
Statin intake, *n* (%)	17 764 (71.5)	24 859
Low dose	255 (1.4)	17 764
Intermediate dose	10 007 (56.3)	17 764
High dose	4727 (26.6)	17 764
Dose unknown	2775 (15.6)	17 764
Ezetimibe^a^ intake, *n* (%)	3548 (14.3)	24 859
PCSK9 inhibitor^a^ intake, *n* (%)	49 (0.2)	24 859
Bempedoic acid^a^ intake, *n* (%)	92 (0.4)	24 859
Fibrate^a^ intake, *n* (%)	915 (3.7)	24 859
Bile acid sequestrants^a^ intake, *n* (%)	85 (0.3)	24 859
Never treated with LLT, *n* (%)	6242 (25.1)	24 859

Abbreviations: ASCVD, atherosclerotic cardiovascular disease; BMI, body mass index; eGFF, estimated glomerular filtration rate; LLT, lipid‐lowering therapy; NYHA, New York Heart Association; PCSK9, proprotein convertase subtilisin kexin 9; SCORE2, Systematic Coronary Risk Estimation 2; TOD, target organ damage.

^a^ Latest treatment.

In this cohort of patients with ASCVD, the mean (± SEM) latest measurement of LDL‐cholesterol patients was 93.8 ± 0.3 mg/dL and the mean (± SEM) of the highest LDL‐cholesterol levels reached per patient in the medical records was 125.5 ± 0.3 mg/dL.

**Figure 1:**
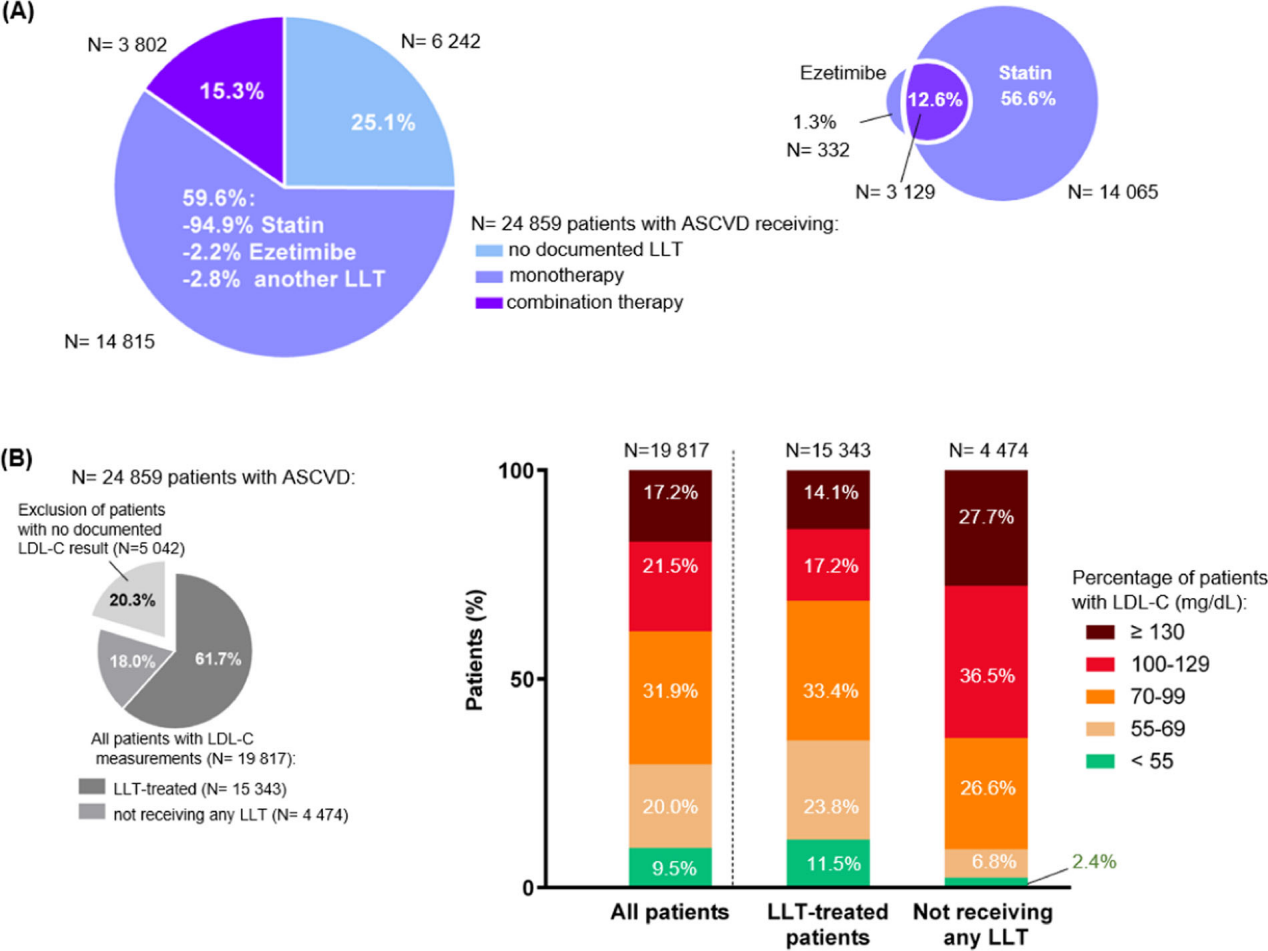
LLT treatment choice in patients with ASCVD and impact on LDL‐C levels. (A) Treatment options in *N* = 24 859 patients with ASCVD (left). Focus on the percentage of patients who were prescribed statins or ezetimibe as monotherapy or as combination therapy (after exclusion of patients receiving non‐statin or non‐ezetimibe LLTs) (right). (B) Among 24 859 patients with ASCVD, 5042 patients were excluded due to the lack of a documented history of LDL‐C testing (left). The 19 817 patients who underwent LDL‐C testing were divided into two groups: 15 343 patients receiving LLT and 4474 patients without any documented LLT, further categorized based on the latest LDL‐C levels available in their medical files (right). Percentages of patients per condition are provided.

Of 24 859 patients, 18 617 patients (74.9%) were prescribed an LLT, with an average treatment duration of 7.6 years (Table 1). Most patients were treated with statins (71.5%), and the dosage received was predominantly intermediate (56.3% of patients treated with statins). A total of 14.3% of these patients were prescribed ezetimibe. A minority of patients were prescribed another LLT (Table 1). Overall, 59.6% of patients were receiving monotherapy (Figure 1A). The most frequently documented LLT was statin monotherapy, used in 56.6% of all patients with ASCVD (14 065 patients). A small proportion (750 patients) was treated with another LLT monotherapy, including 332 patients receiving ezetimibe alone. Combination LLT was used in 15.3% of patients [3802 patients (Figure 1A), of whom 3129 were on combination therapy of ezetimibe and statin (i.e., 82.3% of patients with ASCVD and combination LLT), 87 patients were on ezetimibe and another LLT (0.3% of patients with ASCVD), 570 on statin and another LLT (2.3% of patients with ASCVD), and 16 on another combination LLT]. These results show that most patients treated with ezetimibe were concomitantly treated with statins (i.e., out of 24 859 patients with confirmed ASCVD, 14 065 were receiving statins as monotherapy, 332 ezetimibe as monotherapy, and 3129 patients were treated with statin and ezetimibe, as indicated in Figure 1A). More than one‐third of those treated with LLT experienced a discontinuation of treatment or a switch, as reported by the physician (37.9%; Table 1).

Strikingly, one‐fourth of patients with ASCVD had no documented prescription for LLT (Table 1 and Figure 1A). Further, most untreated ASCVD patients with reported LDL‐C measurement (64.2%) displayed elevated LDL‐C levels ≥ 100 mg/dL (Figure 1B).

To further unravel the link between disease characteristics and treatment choice in the real world, we examined LDL‐C levels in these patients with ASCVD. A total of 19 817 patients had undergone an LDL‐C measurement, as reported in the medical file, including 15 343 (61.7%) LLT‐treated patients (at least one prescription) and 4474 (18.0%) patients who were never prescribed an LLT (Figure 1B). We found a > 3‐fold increase in the proportion of patients attaining LDL‐C < 55 mg/dL or LDL‐C between 55 and 69 mg/dL in those receiving an LLT (11.5% vs. 2.4% of untreated patients, or 23.8% vs. 6.8%, respectively). Among those with no documented LLT, the proportion of patients with LDL‐C ≥ 100 mg/dL was more than twice that of treated patients (36.5% of untreated vs. 17.2% of LLT‐treated patients with LDL‐C between 100 and 129 mg/dL and 27.7% of untreated vs. 14.1% of LLT‐treated patients with LDL‐C ≥ 130 mg/dL). Collectively, the data confirmed the expected benefits of LLTs on LDL‐C levels.

**Figure 2:**
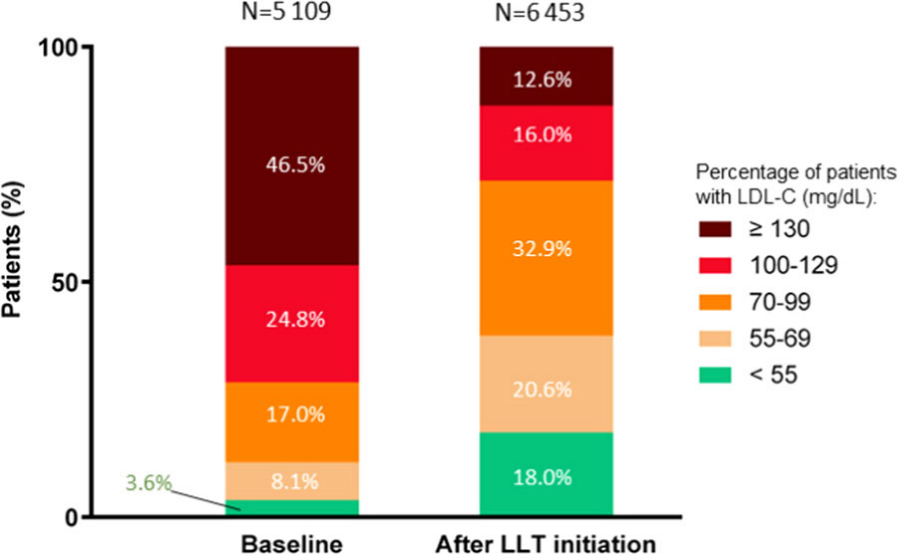
Impact of LLT on the proportion of patients with ASCVD per LDL‐C range. Baseline LDL‐C level was reported in 5109 patients, and LDL‐C level following LLT initiation in 6453 patients. Percentages of patients per LDL‐C range are provided.

We further examined the evolution of LDL‐C levels in the group of 15 343 LLT‐treated patients. A total of 5109 patients had a reported baseline LDL‐C level and 6453 patients had an LDL‐C result following LLT initiation and with LDL‐C value obtained at a mean ± SEM duration of 6.4 years (2341 ± 20 days) after LLT start (Figure 2). We showed that the proportion of patients with LDL‐C levels ≥ 100 mg/dL was decreased by more than half following LLT initiation, with 71.3% of patients falling in this category before LLT initiation versus 28.6% of patients following LLT initiation. Specifically, the proportion of patients with LDL‐C levels ≥ 130 mg/dL was very high (46.5% of patients before vs. 12.6% after LLT start; Figure 2). Before LLT initiation, the proportion of patients with LDL‐C levels between 70 and 99 mg/dL was also almost twice that of treated patients. Following LLT initiation, the proportion of LLT‐treated patients with LDL‐C levels < 70 mg/dL increased by almost fourfold, as compared with those who did not start LLT yet (Figure 2). Despite the improvement, after LLT initiation, only 18% of patients achieve LDL‐C levels of < 55 mg/dL, as recommended by the 2021 ESC guidelines [2].

**Figure 3:**
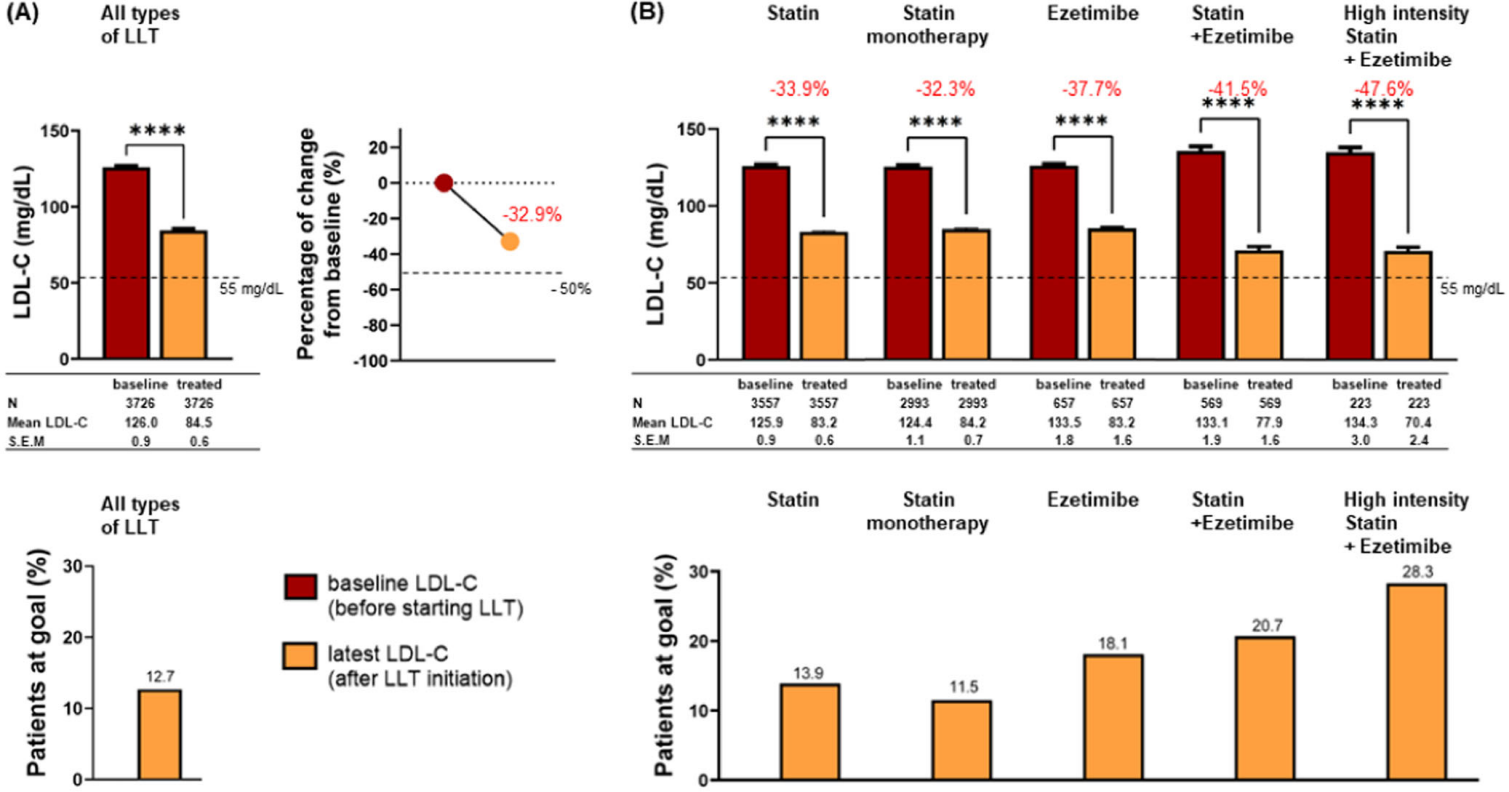
Changes in LDL‐C levels in patients with ASCVD treated with LLT. (A) All LLTs, (B) per type of LLT, (A, B) displaying the latest LLT. (A) Absolute levels, change from baseline (top), and percentage of patients at goal (bottom) in *N* = 3726 patients, that is, all patients with ASCVD having paired values of LDL‐C at baseline versus after LLT initiation (latest values), all LLTs included. (B) Absolute levels (top), and percentage of patients at goal (bottom), with all patients with ASCVD having paired values of LDL‐C at baseline versus after LLT initiation (latest values) per type of LLT. The most frequently documented LLTs are displayed, including from left to right: statin (monotherapy and combination therapy), statin alone (monotherapy), ezetimibe (monotherapy and combination therapy), a combination of ezetimibe and statin (possibly with other LLTs), and high‐intensity statin plus ezetimibe (possibly with other LLTs). A separate group of patients receiving ezetimibe as monotherapy only includes 290 patients with documented LDL‐C levels; therefore, the related data are not represented in this figure. The total number of patients per condition in the database is included in the analysis. The change from baseline is displayed for each group. The number of patients per condition, mean, and SEM is provided in the tables. For absolute levels of LDL‐C, data are represented as mean ± SEM. ****p < 0.0001 versus baseline.

Among patients receiving LLT, there were 3726 patients with paired LDL‐C values at baseline versus following LLT initiation (Figure 3A). The average treatment duration for these patients was 5 years (mean ± SEM duration of 1896 ± 23 days). They were prescribed a mean ± SEM of 1.50 ± 0.01 molecules (per patient) during this period, including a mean ± SEM of 0.35 ± 0.01 up‐titrations (not shown). The treatment with LLT resulted in a significant decrease in LDL‐C levels (reduced by −33%, from 125.96 ± 0.90 to 84.51 ± 0.60 mg/dL; *p* < 0.0001). Despite the improvement in LDL‐C levels, only 12.7% (472/3726) of patients with ASCVD were at goal, as defined by LDL‐C levels < 55 mg/dL and an LDL‐C reduction of ≥ 50% versus baseline (Figure 3A) [2].

**Figure 4:**
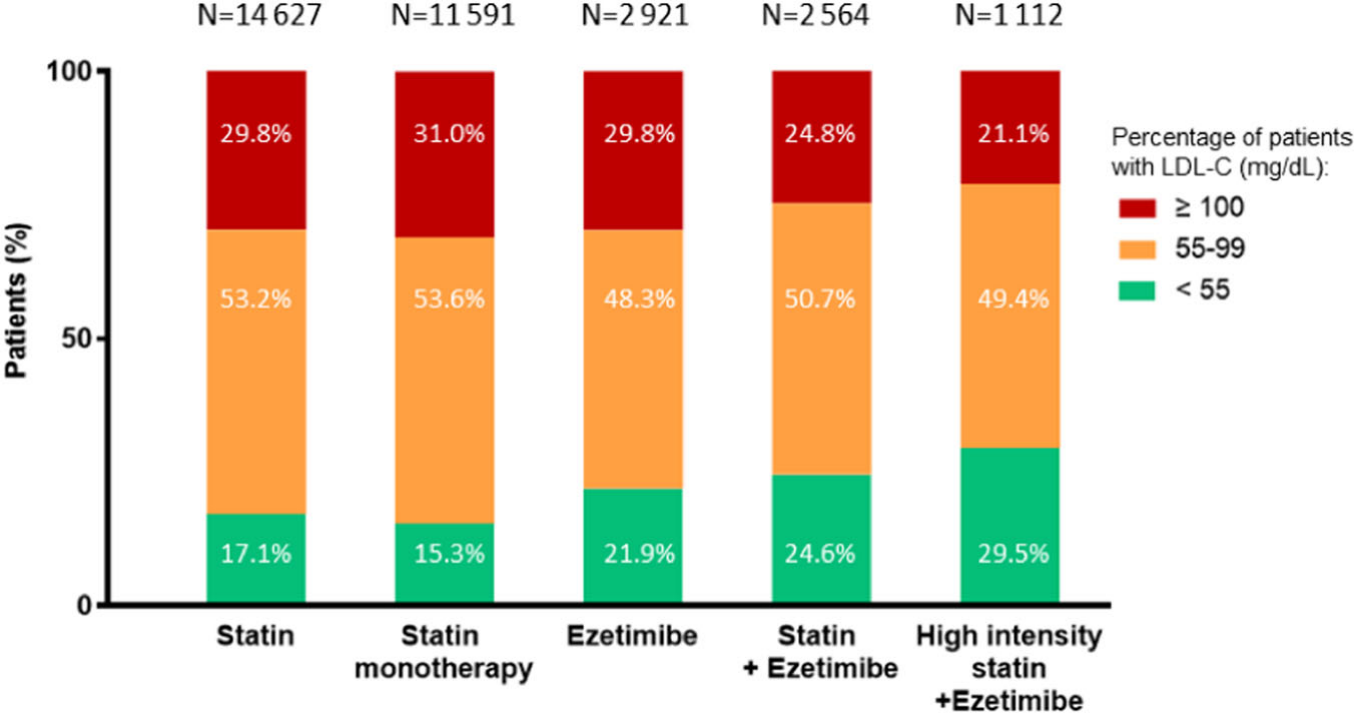
Impact of LLT type on the proportion of patients with ASCVD per LDL‐C range. For each treatment, the percentages of patients per LDL‐C range type are provided, based on their latest LDL‐C levels. The latest LLT is displayed, including from left to right: statin (monotherapy and combination therapy), statin alone (monotherapy), ezetimibe (monotherapy and combination therapy), a combination of ezetimibe and statins (possibly with other LLTs), and high‐intensity statin plus ezetimibe (possibly with other LLTs).

We further examined the proportion of patients with elevated LDL‐C ≥ 100 mg/dL per most frequent LLT treatment category [i.e., patients with recorded history of (i) statin monotherapy and combination therapy, (ii) statin alone (monotherapy), (iii) ezetimibe monotherapy and combination therapy or (iv) ezetimibe and statin combination therapy, with or without other LLTs, and (v) high‐intensity statin plus ezetimibe, with or without additional LLTs] (Figure 4). The groups having the smallest proportion of patients with LDL‐C ≥ 100 mg/dL were those receiving combination therapy including ezetimibe and statins (24.8% of patients treated with statin plus ezetimibe, but this percentage decreased to 21.1% in the group receiving high‐intensity statin plus ezetimibe, vs. ≥ 29.8% of patients with other therapies falling in this category; Figure 4). Consistently, the patients treated with combination therapy including ezetimibe and statins exhibited the greatest improvements in LDL‐C levels, with LDL‐C levels reduced by −41.5% from baseline and reaching −47.5% with high‐intensity statin plus ezetimibe (*p* < 0.0001; Figure 3B). Nevertheless, only 20.7% of those with statin plus ezetimibe and 28.3% of those with high‐intensity statin plus ezetimibe were at goal (Figure 3B).

As per recommended guidelines for patients with ASCVD [2], LDL‐C goals should be reached with a maximum tolerated dose of statin and ezetimibe and intensified with a PCSK9 inhibitor when required. In Belgium, certain PCSK9 inhibitors are reimbursed for patients with established ASCVD and with LDL‐C ≥ 100 mg/dL despite being treated with a maximally tolerated dose of statin and ezetimibe [17, 18]. As described in Table 1, there was only a minority of patients with reported use of PCSK9 inhibitors in this cohort (49 patients only). Yet, the LDL‐C reduction observed with PCSK9 inhibitors was qualitatively similar to that achieved with high‐intensity statin plus ezetimibe, demonstrating the benefit of this therapy (not shown).

## 4 Discussion

Among the 40 888 (14.4%) patients with very high CV risk, 24 859 had established ASCVD. With this result, we evaluated that ~967 000 patients are living with ASCVD in Belgium, which is higher than the number recently estimated based on the prevalence of the most important clinical outcomes of ASCVD (~750 000 patients) [13]. We found that most patients with ASCVD were either receiving only monotherapy (59.6%) or had no documented LLT (25.1%). Further, 64.2% of those with no documented LLT exhibited LDL‐C levels ≥ 100 mg/dL. Collectively, the picture of the lipid management landscape in Belgium seems more concerning than that recently described at the European level, in which 17.8% of patients with high or very high CV risks had no LLT, 59.6% were receiving monotherapy, and 22.7% were using combination therapy, explained by the underestimation of the CV risk by some physicians [10]. The difference might be explained by the data collected from GP practices in the present study, while the insights into the management of LLT in Europe involve multiple care settings (primary care, secondary care, and different specialties) [10]. The small number of patients with pre‐ versus posttreatment LDL‐C testing further illustrates the inadequate physician‐led lipid management.

Our analysis shows that overall, using LLT significantly reduces LDL‐C concentrations by 33% in patients with ASCVD. The addition of non‐statin LLT (combination therapies) leads to the greatest impact. Among common treatment options, a reduction of LDL‐C levels by 41.5% was achieved with combination therapy including statin and ezetimibe, and by 47.6% with high‐intensity statin plus ezetimibe. Yet, in these groups, 24.8%–21.1% of patients had still elevated LDL‐C levels ≥ 100 mg/dL, respectively, and only 20.7%–28.3% were at goal.

Patients receiving PCSK9 inhibitors achieved high reductions of LDL‐C levels (not shown), and thus, lower risk of CV events. However, the number of patients receiving PCSK9 inhibitors was very limited, due to the restrictive reimbursement conditions (for details see references [17, 18, 19]), and might impair the ability to extrapolate these results to larger populations.

Additional investigation is needed to comprehend the underlying reasons for the high proportion of ASCVD patients with no documented LLT (despite LDL‐C level ≥ 100 mg/dL) or only receiving monotherapy. In addition to this insufficient management of dyslipidemia, we further show that one‐third of the patients with ASCVD using an LLT experienced an interruption in their treatment. Despite their clear benefits, long‐term adherence to therapies for cardiometabolic disorders, including LLT, remains low [20, 21, 22], even among patients with a history of CV events [23]. In this database, when a medical reason was reported by the physician, health improvement accounted for ~33% of LLT interruptions (Figure S3). However, lack of efficacy and intolerance or contraindication were attributed to ~67% of the interruptions (Figure S3), suggesting that the journey might be challenging for both healthcare professionals and patients.

## 5 Conclusions

Collectively, our results illustrate that lipid management in patients with ASCVD remains suboptimal. Our study underscores the necessity for increased awareness about the adverse impacts of prolonged exposure to high LDL‐C on CV health. Our study emphasizes the need to elaborate strategies to assist patients in achieving their LDL‐C goals, with a particular focus on supporting the implementation of intensification of LLT in routine clinical practice. Given the nature of our study as an observational and noninterventional investigation, the patients had full autonomy to navigate their adherence to medication, lifestyle choices, and management of risk factors through routine care.

### 5.1 Limitations

The limitations of this analysis should be considered. Our findings are based on the analysis of databases limited to generalists in Belgium and might include imprecisions in reporting. An example is the very high prevalence of heterozygous familial hypercholesterolemia in this study, in comparison with other European data sources [24], suggesting that some comorbidities may be misdiagnosed. Therefore, our findings may not be generalizable to the overall population. Moreover, big databases have limitations such as punctual errors in reporting or missing values, but these issues are expected to be mitigated by the large number of patients.

## Author Contributions

Concept: M.J. Conduct: E.M., S.B., and M.J. Data analysis: S.B. and E.M. Interpretation: all authors. Writing: E.M. (draft) and all authors (revision).

## Ethics Statement

Data collection and analysis were approved by the Belgian Data Protection Authority. The THIN database consists of anonymized electronic medical records compliant with the European general data protection regulations. The Belgian legislation did not require additional ethical approval.

## Consent

The need for informed consent was deemed unnecessary according to national regulations (Belgian Data Protection Authority).

## Conflicts of Interest

Eléonore Maury and Mieke Jansen are employees of the sponsor. Samuel Brouyère is an employee of Cegedim Health Data Belgium and received consulting fees from Novartis during the conduct of this study.

## Supporting information

Supporting information.

## Data Availability

Additional information is available from the corresponding author (eleonore.maury@novartis.com) upon reasonable request. Raw data (at the patient level) underlying this article may not be transferred due to ethical reasons and security considerations.
