# Supplementary material for: Characteristics of Patients With Atherosclerotic Cardiovascular Disease in Belgium and Current Treatment Patterns for the Management of Elevated LDL‐C Levels
Source: Clin Cardiol. 2024 Aug 29;47(9):e24330. doi: 10.1002/clc.24330 (PMC11358763; doi:10.1002/clc.24330)

**Supplementary material**

**Supplementary methods:**

THIN^®^ is a private database relying on anonymized patient data from general practices. Anonymized medical records collected at the healthcare professional-level are coded according to the International Statistical Classification of Diseases and Related Health Problems, Tenth Revision (ICD-10). A representative panel of over 300 general practitioners (GPs) were contracted for their contribution to the electronic data capture. A third party is responsible for adding another layer of encryption to the anonymized health records and for the subsequent daily upload of the data into the THIN^®^ database. The distribution of the medical workforce contributing to the THIN^®^ database^1^ is consistent with the latest HWF STATAN report^2^ and includes >35 GPs located in Brussels capital region, >175 in Flanders and >95 in Wallonia. In Belgium, THIN^®^ relies on the patient management software Health One^®^, which is used by over 2 600 healthcare professionals (HCPs) and is compliant with local regulations. The software optimizes patient management, reporting prescribed medications or lab tests and registering biometrics in a centralized platform.

As a data quality control measure, practices were eligible for inclusion in this study only if physicians had i) a recorded participation of at least 1 year before November-2019 and ii) work interruptions shorter than 1 month per year between November-2019 and October-2022.

No formal medical examinations or laboratory tests were mandated as part of this non-interventional observational study.

Previous or current cardiometabolic disease were determined based on the following ICD10 diagnosis: F01.9, G45-G46, G97.2, I20-I25, I34.0, I60-I69, I70-I79, Z50.0, ischemic stroke, percutaneous coronary intervention (PCI), coronary artery bypass grafting (CABG), thromboendarterectomy, evidence of chronic kidney disease (based on estimated Glomerular Filtration Rate, eGFR) and renal dysfunction (based on ICD10: N00-N19, N25-N29), type 1 or type 2 diabetes and consequences (based on ICD10: E10-E14, E74.8, R73.0, G59.0, G63.2, H28.0, H36.0, M14.2, N08.3)]. All patients with medical data recorded one year or more before their first data during this timeframe were selected. There were no other exclusion criteria.

The data extraction and cohort selection were facilitated using structured query language (SQL) coding SQL Server Express 15.0, MS SQL Server Management Studio 18.12 and Python 3.9.16.

Based on the estimated number of patients at the national scale, a projection factor of 38.9 was used to evaluate the total number of patients living with ASCVD in Belgium.

**Supplementary references:**

1. The Health Improvement Network. https://www.cegedim-health-data.com/cegedim-health-data/thin-the-health-improvement-network/. Accessed December 2022
2. HWF STATAN 2020. https://organesdeconcertation.sante.belgique.be/fr/documents/hwf-statan-2020-statistiques-detaillees**,** Accessed December 2022

**Supplementary results:**

**Figure S1. Classification of the patients based on CV risk.** Among 283 700 patients (who underwent a medical examination at a GP practice between Nov2019-Oct2022 and with at least 1 year of historical data), 173 989 patients had no recorded CV risk factors, and 109 711 patients were classified into low–moderate risk, high risk, and very high risk. Within these subgroups, each patient could fall into several subcategories. Absolute number of patients per condition and related percentage are provided.


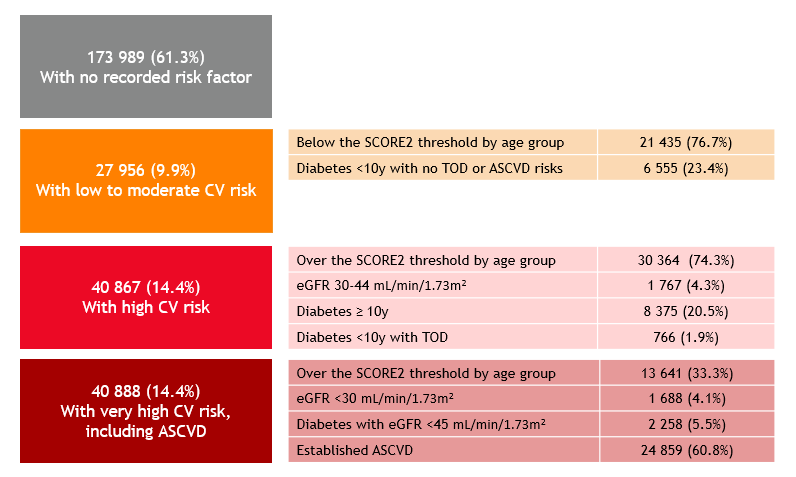


Abbreviations: Atherosclerotic cardiovascular disease (ASCVD), Cardiovascular risk (CV risk), Systematic Coronary Risk Estimation 2 (SCORE 2), estimated glomerular filtration rate (eGFF), target organ damage (TOD).

**Figure S2. Evolution of CV risk from low-moderate to high and very high.** For the 283 700 patients (with at least 1 year of historical data), the evolution was determined based on the most severe status at any time, with, at max., full history available since 2005. Hence, the evolution is based on at least 1 year as per inclusion rules, and with a maximum of 17 years.


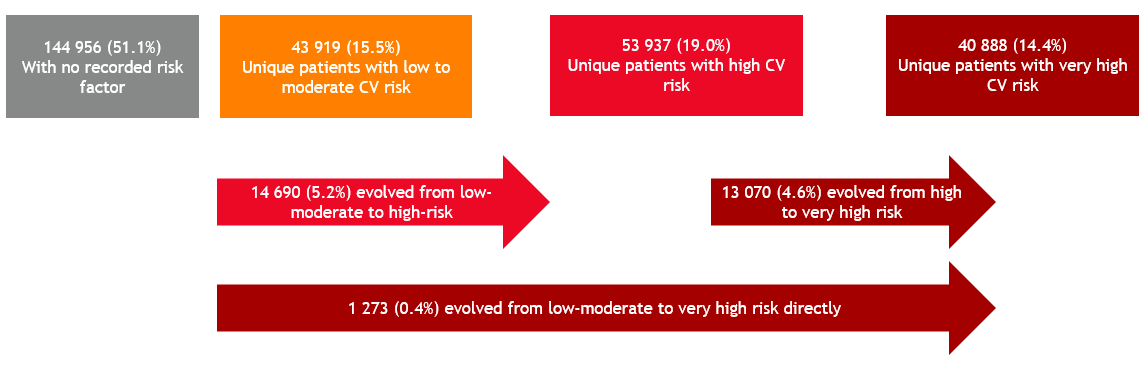


Abbreviation: Cardiovascular risk (CV risk).

**Figure S3. Medical reason for LLT interruption in Belgium.** Data from all patients included in the database and with history of LLT, not limited to very high CV risk or ASCVD, were analyzed (N=19 716 patients).


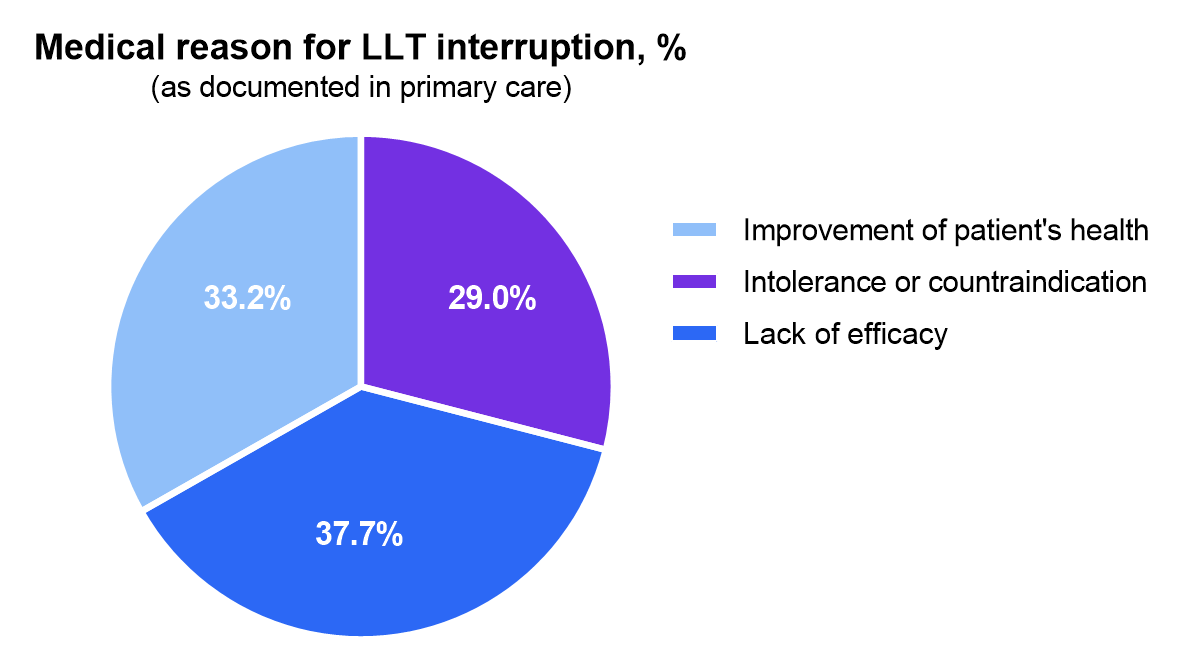

Supplement: Supplementary file 1 — Supporting information. [file CLC-47-e24330-s001.docx]
